# Supplementary material for: Integrated multimodal artificial intelligence framework for healthcare applications
Source: NPJ Digit Med. 2022 Sep 20;5:149. doi: 10.1038/s41746-022-00689-4 (PMC9489871; doi:10.1038/s41746-022-00689-4)
Supplement: Supplementary file 1 — Supplemental Materials [file 41746_2022_689_MOESM1_ESM.pdf]

SUPPLEMENTAL MATERIAL

Integrated multimodal artificial intelligence framework for healthcare applications

Luis R. Soenksen[1,4*], Yu Ma[2*], Cynthia Zeng[2*], Leonard D.J. Boussioux[2*], Kimberly M Villalobos[2*], Liangyuan Na[2*], Holly Mika Wiberg[2], Michael L. Li[2], Ignacio Fuentes[1], Dimitris Bertsimas[1,2,3] ‡

[1]Abdul Latif Jameel Clinic for Machine Learning in Health, MIT, Cambridge, MA 02139, USA. [2]Operations Research Center, Massachusetts Institute of Technology (MIT), Cambridge, MA 02139, USA. [3]Sloan School of Management, MIT, Cambridge, MA 02139, USA. [4]Wyss Institute for Biologically Inspired Engineering, Harvard University, Boston, MA 02115, USA.

* These authors contributed equally to this work

‡ Corresponding author. Email: dbertsim@mit.edu

Supplemental Tables

#	Chart events	Laboratory events	Procedure events
1	Heart rate	Glucose	Foley Catheter
2	Non-invasive systolic blood pressure	Potassium	PICC Line
3	Non-invasive blood diastolic pressure	Sodium	Intubation
4	Non-invasive nominal blood pressure	Chloride	Peritoneal dialysis
5	Respiratory rate	Creatinine	Bronchoscopy
6	O_2 saturation by pulse oximetry	Urea nitrogen	EEG
7	Verbal GCS response	Bicarbonate	Dialysis CRRT
8	Eye opening GCS response	Anion gap	Dialysis catheter
9	Motor GCS response	Hemoglobin	Chest tube removed
10		Hematocrit	Hemodialysis
11		Magnesium	
12		Platelet count	
13		Phosphate	
14		White Blood Cells	
15		Total calcium	
16		MCH	
17		Red Blood Cells	
18		MCHC	
19		MCV	
20		RDW	
21		Platelet count	
22		Neutrophils	
23		Vancomycin	

Supplemental Table 1. Patient signals in HAIM-MIMIC-MM by type of event used as time-series for embedding extraction. Nine time-dependent signals were derived from procedures, twenty-three were derived from laboratories, and eight were derived from information included in the patient chart. CRRT=Continuous renal replacement therapy, EEG=Electroencephalogram, GCS=Glasgow Coma Scale, MCH=Mean corpuscular hemoglobin, MCHC=Mean corpuscular hemoglobin concentration, PICC=Peripherally inserted central catheter, RDW=Red blood cell distribution width.

#	Data Modalities	#	Data Sources	Extracted Features
1	Tabular	1	Demographics (E_{de})	6
2	Time-series	2	Chart events ($E_{ce)}$	99
		3	Laboratory events (E_{le})	242
		4	Procedure events (E_{pe})	110
3	Text	5	Radiological notes (E_{radn})	768
		6	Electrocardiogram notes (E_{ecgn})	768
		7	Echocardiogram notes (E_{econ})	768
4	Images	8	Visual probabilities (E_{vp})	18
		9	Visual dense-layer feature (E_{vd})	18
		10	Aggregated visual probabilities (E_{vmp})	1024
		11	Aggregated visual dense-layer features (E_{vmd})	1024

Supplemental Table 2. List of different data modalities and data sources used to test the HAIM framework based on the HAIM-MIMIC-MM database. There are a total of four data modalities and eleven data sources. All data sources correspond to only one data modality. Thus, a model trained on a single data modality can have as little as 1 data source and many as 4 different data sources (of the same kind) as inputs. Double, triple and quadruple modality models can have a number of data sources ranging from [2 to 7], [3 to 9] and [4 to 11], respectively. The number of features (per data source) extracted by the pre-trained feature extractors selected for our demonstration of the HAIM pipeline based on HAIM-MIMIC-MM are also shown.

Feature Name	Missing %	Source	Handling
anchor_age	0.0	Demographics	N/A
gender_int	0.0	Demographics	N/A
ethnicity_int	0.0	Demographics	N/A
marital_status_int	0.0	Demographics	N/A
language_int	0.0	Demographics	N/A
insurance_int	0.0	Demographics	N/A
Foley Catheter	82.6	Procedure	Fill with 0
PICC Line	63.7	Procedure	Fill with 0
Intubation	75.3	Procedure	Fill with 0
Peritoneal Dialysis	99.7	Procedure	Fill with 0
Bronchoscopy	81.5	Procedure	Fill with 0
EEG	91.5	Procedure	Fill with 0
Dialysis – CRRT	93.1	Procedure	Fill with 0
Dialysis Catheter	88.9	Procedure	Fill with 0
Chest Tube Removed	93.1	Procedure	Fill with 0
Hemodialysis	92.9	Procedure	Fill with 0
Glucose	4.4	Lab	Fill with 0
Potassium	4.7	Lab	Fill with 0
Sodium	4.7	Lab	Fill with 0
Chloride	4.7	Lab	Fill with 0
Creatinine	4.7	Lab	Fill with 0
Urea Nitrogen	4.7	Lab	Fill with 0
Bicarbonate	4.7	Lab	Fill with 0
Anion Gap	4.7	Lab	Fill with 0
Hemoglobin	4.7	Lab	Fill with 0
Hematocrit	4.8	Lab	Fill with 0
Magnesium	5.4	Lab	Fill with 0
Platelet Count	9.8	Lab	Fill with 0
Phosphate	6.0	Lab	Fill with 0
White Blood Cells	4.9	Lab	Fill with 0
Calcium, Total	6.0	Lab	Fill with 0
MCH	4.9	Lab	Fill with 0
Red Blood Cells	4.9	Lab	Fill with 0
MCHC	4.9	Lab	Fill with 0
MCV	4.9	Lab	Fill with 0
RDW	4.9	Lab	Fill with 0
Neutrophils	36.9	Lab	Fill with 0
Vancomycin	60.0	Lab	Fill with 0
Heart Rate	19.5	Chart	Fill with 0
Non-Invasive Blood Pressure systolic	23.4	Chart	Fill with 0
Non-Invasive Blood Pressure diastolic	23.4	Chart	Fill with 0
Non-Invasive Blood Pressure mean	23.3	Chart	Fill with 0
Respiratory Rate	19.5	Chart	Fill with 0
O2 saturation pulse oximetry	19.6	Chart	Fill with 0
GCS - Verbal Response	20.8	Chart	Fill with 0

GCS - Eye Opening	20.7	Chart	Fill with 0
GCS - Motor Response	20.8	Chart	Fill with 0
Electrocardiogram Notes	11.2	Notes	Empty String
Echocardiogram Notes	30.5	Notes	Empty String
Radiology Notes	0.1	Notes	Empty String

Supplemental Table 3. List of missing data percentages by individual variables and handling strategy. Individual variables (i.e., feature name) within key HAIM-MIMIC-MM data source groups are shown. The strategy for missing value handling used in our tests is as follows: 1) We exclude patients with no available X-rays from our selection cohort; 2)Time-series features are imputed with 0 if there is no measurement at any timestamp; 3)Text embeddings are generated on from an empty string if there is no note available; 4)There were no missing values for demographics data.

Supplemental Figures

Supplemental Fig. 1. Compilation of performance metrics across sample trained models on HAIM-MIMIC-MM A) Values of area under the receiver operating characteristic (AUROC) curves and B) Standard deviations, for all models trained for the pathology diagnosis tasks (i.e., lung lesions, fractures, atelectasis, lung opacities, pneumothorax, enlarged cardio mediastinum, cardiomegaly, pneumonia, consolidation, and edema), ordered by individual combinations of the used 10 input sources (total=1,023 models). C) Values of AUROC curves and D) Standard deviations for length-of-stay and 48-hour mortality prediction tasks), ordered by individual combinations of the used 11 inputs sources (total=2,047 models). Additional input source in length-of-stay and 48-hour mortality prediction tasks corresponds to radiology notes, which were not used in the pathology diagnosis tasks to prevent overfitting or misrepresentation of predictive capacity of trained models.

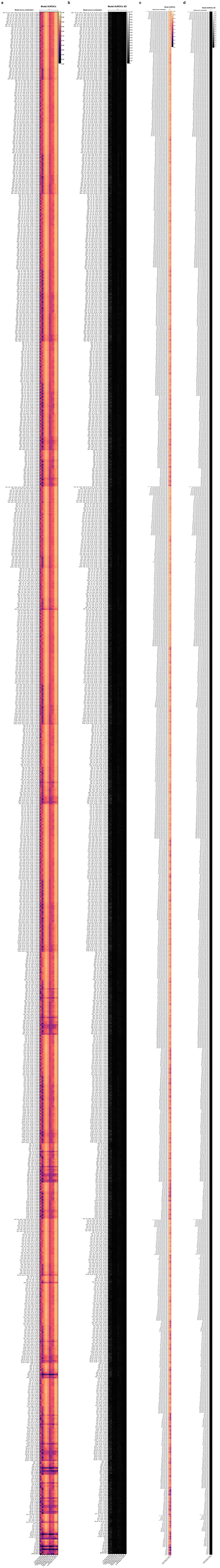
a
Model AUROCs
b
Model AUROCs SD
c
d

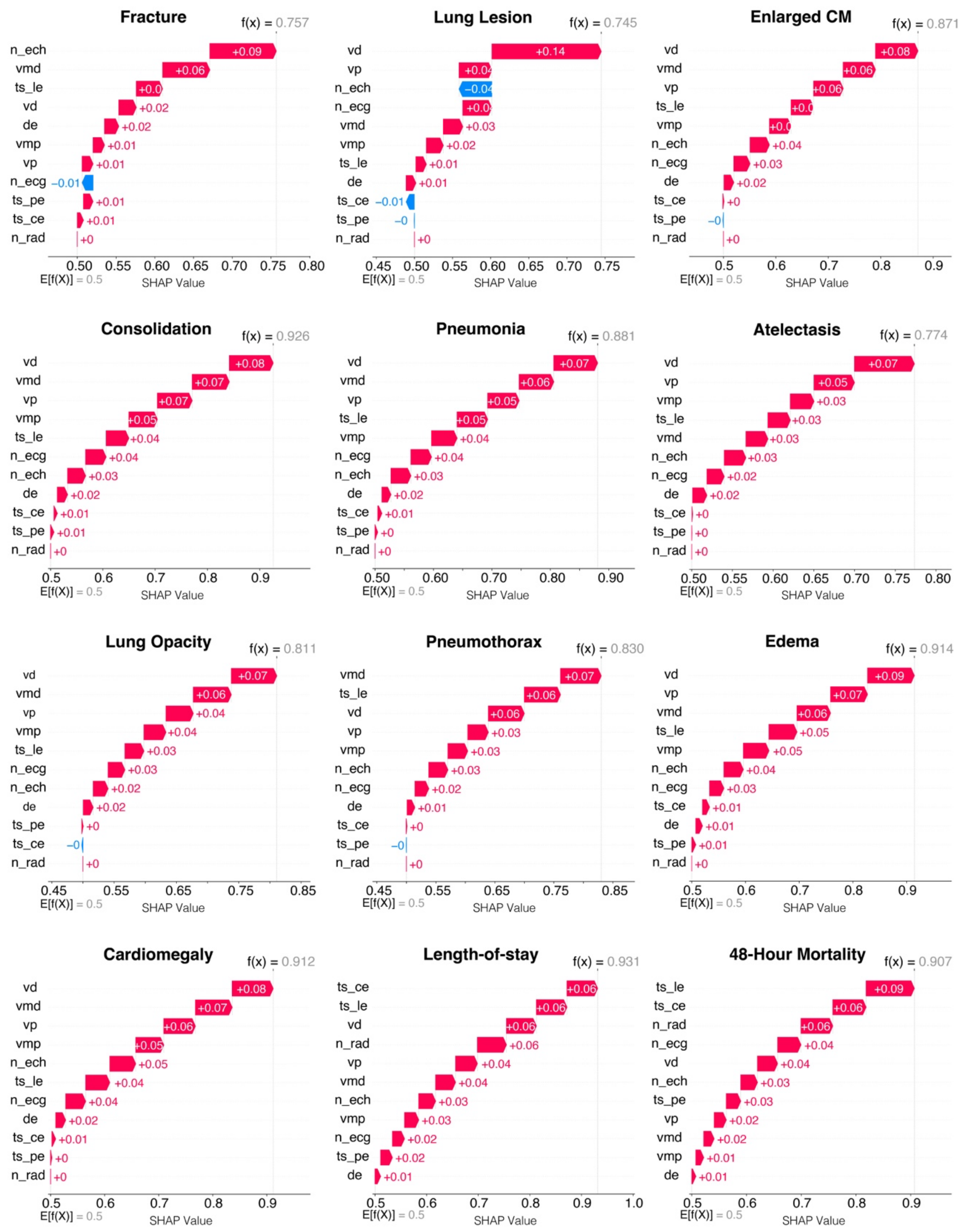

Supplemental Fig. 2. Waterfall plots of aggregated Shapley values for independent data sources per predictive task. Shapley values for different tasks exhibit distinct distributions of aggregated Shapley values across input data sources, with mostly positively contributing effects towards predictive capacity (red values pointing right), with the exception of a small number of Shapley values with marginal negative values (blue values pointing left).

Algorithm 1 Holistic Artificial Intelligence in Medicine (HAIM) Experiments Pipeline

Input:
$\mathcal{I}$: Set of data modalities
$\mathcal{K}$: Set of prediction tasks
$\mathcal{P}$: Set of hyperparameter combinations
$\mathcal{M}$: Set of evaluation metrics
$\boldsymbol{X}^i, f_i$: Feature data and feature extractor for data modality i
$\boldsymbol{y}_k$: Target vector for task k
g_p: Predictive model with parameter p
L_k: Loss function for task k
s: Number of random seeds to reproduce experiments
c: Number of cross-validation folds
Remarks: $|\cdot|$ denotes the cardinality of a set and [n] denotes the set $\{1, 2, ..., n\}$ for any positive integer $n \in \mathbb{N}$.

Output:
$\boldsymbol{E}$: One-dimensional HAIM embedding
$g_k^* \quad \forall k \in \mathcal{K}$: Trained HAIM models with highest observed performance on prediction tasks $k \in \mathcal{K}$
Performance results: Performance metrics for $\mathcal{M}$ per trained HAIM model

for $i \in \mathcal{I}$ **do**
  $\boldsymbol{e}^i \leftarrow f_i(\boldsymbol{X}^i)$ ▷ Generate embeddings of source i using SOTA model f_i
  $\boldsymbol{e}_v^i \leftarrow \text{vec}(\boldsymbol{e}^i)$ ▷ Flatten vector $\boldsymbol{e}^i$ to be one-dimensional
  $\boldsymbol{e}_n^i \leftarrow \frac{\boldsymbol{e}_v^i - \min(\boldsymbol{e}_v^i)}{\max(\boldsymbol{e}_v^i) - \min(\boldsymbol{e}_v^i)}$ ▷ Normalize vector $\boldsymbol{e}_v^i$ by min-max scaling
end for
$\boldsymbol{E} \leftarrow [\boldsymbol{e}_n^1, \boldsymbol{e}_n^1, \ldots, \boldsymbol{e}_n^{|\mathcal{I}|}]$. ▷ Concatenate all flattened and normalized feature embeddings e_n^i into a single one-dimensional HAIM embedding $\mathbf{E}$

for $k \in \mathcal{K}$ **do**
  for $t \in [s]$ **do**
    $\boldsymbol{E}_{train}^t, \boldsymbol{E}_{test}^t, \boldsymbol{y}_{k,train}^t, \boldsymbol{y}_{k,test}^t \leftarrow \text{train\_test\_split}(\boldsymbol{E}, \boldsymbol{y}_k, \text{seed} = t)$
    $g_k^* \leftarrow \text{argmin}_{g_p \forall p \in \mathcal{P}} L(g_p(\boldsymbol{E}_{\text{train}}^t), \boldsymbol{y}_{k,train}^t)$ ▷ c-Fold cross-validation with a grid-search to select best $p^* \in \mathcal{P}$ on the training data
  end for
  Performance results $\leftarrow m(g_k^*(\boldsymbol{E}_{test}^t), \boldsymbol{y}_{k,test}^t) \quad \forall t \in [s], \forall m \in \mathcal{M}$
▷ Evaluate trained HAIM models on test data per seed s
▷ Report average performance of evaluated HAIM models across s seeds
end for

Supplemental Fig. 3. Algorithmic formulation of the HAIM framework. Heterogeneous input data (i.e., tabular, time-series, text, and images) are processed by externally validated state-of-the-art pre-trained models, specific to each data modality, used as feature extractors. The extracted features are represented as vector embeddings e^i that can be easily concatenated into a single normalized vector of fixed dimensionality (HAIM Embedding). Finally, we use this HAIM embedding (E) as input to train a downstream model for each prediction task of interest (g_k^*). SOTA= state-of-the-art.

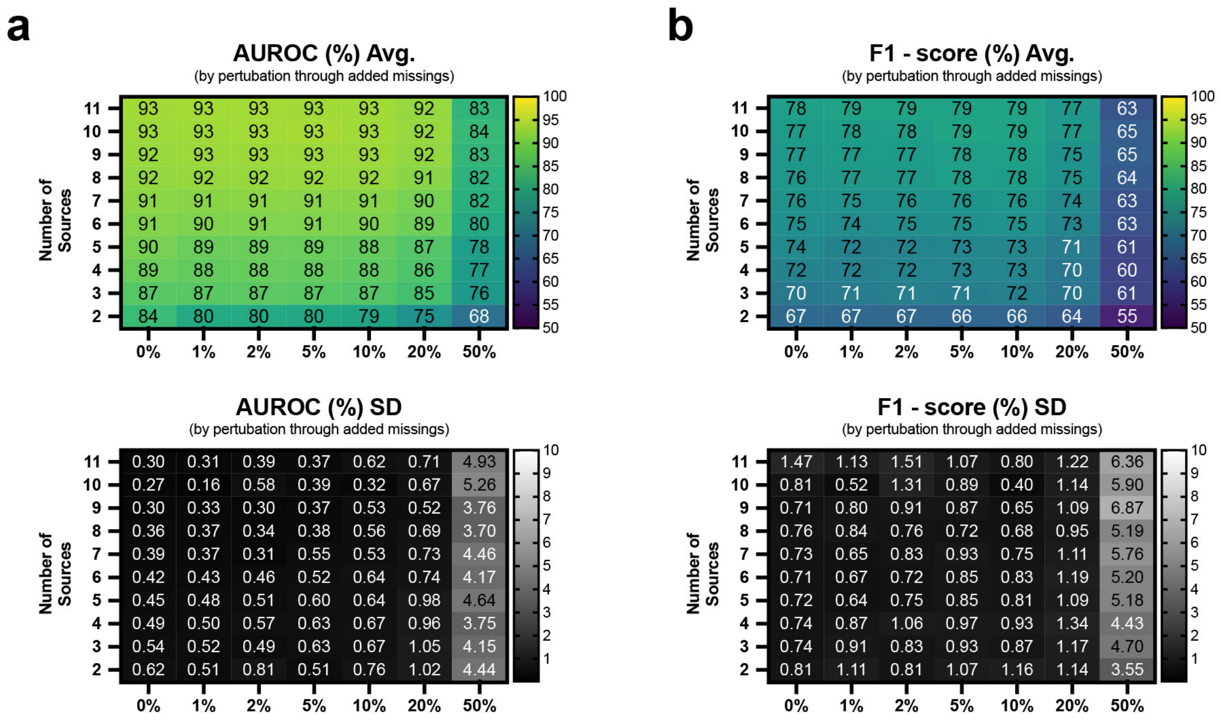

Supplemental Fig. 4. Measured performance on length-of-stay predictions from using versions of HAIM-MIMIC-MM where the demographics data had been synthetically degraded to show the effects of increasing percentages of missing data in this input. To achieve this, we randomly selected 1%, 2%, 5%, 10%, 20%, and 50% of all demographic's features in patient records and substituted them with zeros to emulate different scenarios of missing data. These altered datasets were then used to extract HAIM embeddings and generate downstream predictive models based on our framework. The average and standard deviation of the AUROC curves (A), as well as F1-score (B), were evaluated over the testing set (20%) for five consecutive iterations of randomized train-test data splitting and model training. The range of sources for this test is 2 to 11, as models were trained using demographics data (i.e., tabular) with added missing percentages along with at least one more data source from other modalities (i.e., time-series, text, or images). These results show decreasing sensitivity to missing values as more data sources and modalities are added. Generally robust performance of the models was observed in the presence of missing percentages equal to or below 20% for the tabular data modality. A more noticeable reduction in the performance of trained models was measured in the presence of over 50% missing data in the tabula modality of the patient records. AUROC=area under the receiver operating characteristic; Avg=Average; SD=Standard Deviation.
